# An Antarctic Extreme Halophile and Its Polyextremophilic Enzyme: Effects of Perchlorate Salts

**DOI:** 10.1089/ast.2017.1766

**Published:** 2018-04-01

**Authors:** Victoria J. Laye, Shiladitya DasSarma

**Affiliations:** University of Maryland School of Medicine, Institute of Marine and Environmental Technology, Baltimore, Maryland.

## Abstract

Effects of perchlorate salts prevalent on the surface of Mars are of significant interest to astrobiology from the perspective of potential life on the Red Planet. *Halorubrum lacusprofundi,* a cold-adapted halophilic Antarctic archaeon, was able to grow anaerobically on 0.04 *M* concentration of perchlorate. With increasing concentrations of perchlorate, growth was inhibited, with half-maximal growth rate in *ca*. 0.3 *M* NaClO_4_ and 0.1 *M* Mg(ClO_4_)_2_ under aerobic conditions. Magnesium ions were also inhibitory for growth, but at considerably higher concentrations, with half-maximal growth rate above 1 *M*. For a purified halophilic β-galactosidase enzyme of *H. lacusprofundi* expressed in *Halobacterium* sp. NRC-1, 50% inhibition of catalytic activity was observed at 0.88 *M* NaClO_4_ and 0.13 *M* Mg(ClO_4_)_2_. Magnesium ions were a more potent inhibitor of the enzyme than of cell growth. Steady-state kinetic analysis showed that Mg(ClO_4_)_2_ acts as a mixed inhibitor (*K*_I_ = 0.04 *M*), with magnesium alone being a competitive inhibitor (*K*_I_ = 0.3 *M*) and perchlorate alone acting as a very weak noncompetitive inhibitor (*K*_I_ = 2 *M*). Based on the estimated concentrations of perchlorate salts on the surface of Mars, our results show that neither sodium nor magnesium perchlorates would significantly inhibit growth and enzyme activity of halophiles. This is the first study of perchlorate effects on a purified enzyme. Key Words: Halophilic archaea—Perchlorate—Enzyme inhibition—Magnesium. Astrobiology 18, 412–418.

## 1. Introduction

Halophiles have long been proposed as candidates for survival on Mars since they have evolved to grow in high salt concentrations and multiple extreme conditions on Earth (Landis, 2001; DasSarma, 2006). Their ability to grow in hypersaline environments requires adaptation to a number of other stressors ranging from toxic ions, periods of desiccation, and UV and ionizing radiation (Oren *et al.,* 2014; DasSarma and DasSarma, 2017). A few halophiles, including halophilic archaea, are also adapted to cold temperatures, including subzero temperatures, with the freezing point of water depressed by high salinity (Reid *et al.,* 2006). As a result, these extremophilic microbes may represent potential models for life on Mars where these stressors are commonly found.

Over the past 10 years, the chemical composition on the surface of Mars has been increasingly probed, both remotely and by rovers on site. The presence of oxidizers was first suspected during the NASA Viking lander missions of the 1970s (Klein, 1978). This was later confirmed by the Phoenix lander mission where perchlorate concentrations of 0.4–0.6 wt % (3.3 m*M* Mg^2+^, 2.4 m*M* ClO_4_^−^) were detected by the onboard Wet Chemistry Lab (Hecht *et al.,* 2009) and confirmed by the Sample Analysis at Mars instrument on the Mars Curiosity rover (Glavin *et al.,* 2013). Remote spectral analysis of Palikir Crater and Hale Crater showed the potential for existence of hydrated salts rather than pure water in recurring slope lineae, consistent with magnesium perchlorate. The Horowitz Crater had larger recurring slope lineae indicative of the potential for liquid water and spectra consistent with martian soil mixed with sodium perchlorate (Ojha *et al.,* 2015).

From the perspective of possible survival on Mars, *Halorubrum lacusprofundi* is a halophilic archaeon of significant interest (Reid *et al.,* 2006; DasSarma *et al.,* 2017). This microorganism was isolated from Deep Lake, Antarctica, which is perennially cold and hypersaline (Franzmann *et al.,* 1988). *Halorubrum lacusprofundi*, a polyextremophile, is able to survive under both low temperatures and hypersaline conditions, with measurable growth down to −1°C in medium containing 3.1 *M* NaCl and 0.4 *M* MgCl_2_ (Reid *et al.,* 2006). The genome of *H. lacusprofundi* has been completely sequenced and its derived protein sequences analyzed bioinformatically (DasSarma *et al.,* 2013; Anderson *et al.,* 2016). Like other halophilic archaea, *H. lacusprofundi* proteins are negatively supercharged, which provides both mutual repulsion and enhanced hydration for increased solubility (Karan *et al.,* 2012; DasSarma and DasSarma, 2015). Its conserved protein sequences were found to exhibit slightly reduced negativity at the surface compared to mesophilic, halophilic proteins, and perturbation of internal residues, correlated with enhanced function at cold, hypersaline conditions (DasSarma *et al.,* 2013).

A polyextremophilic β-galactosidase enzyme of *H. lacusprofundi* was studied in detail as a model enzyme for its evolution to extreme conditions (Karan *et al.,* 2013). The *H. lacusprofundi* protein was overexpressed in a related halophile and purified to homogeneity, and characterized for its temperature, pH, and salt optima. Steady-state kinetic analysis was also carried out and provided insights into its biochemical properties, which are similar to other cold-adapted enzymes (Laye *et al.,* 2017). A homology model of the enzyme was also constructed by using a related β-galactosidase enzyme from *Thermus thermophilus,* with structural comparisons showing that the *H. lacusprofundi* protein has much greater surface negative charges than the thermophilic protein (−65 versus −4) (DasSarma *et al.,* 2013).

To determine the effects of martian salts on *H. lacusprofundi,* we subjected the microbe and its enzyme to increasing concentrations of sodium and magnesium perchlorate. Our results show that this polyextremophilic halophile and its model enzyme, while exhibiting sensitivity to these ions, retain their ability to function in their presence at high concentrations far above what is likely to be encountered on the surface of our sister planet.

## 2. Materials and Methods

### 2.1. Culturing

For aerobic growth, cultures of halophiles were shaken in the light in test tubes at 42°C in CM^+^ for *Halobacterium* sp. NRC-1 and at 37°C in ATCC 1682 media for *H. lacusprofundi* (Berquist *et al.,* 2007), adjusted to the desired concentration of NaClO_4_, MgCl_2_, and Mg(ClO_4_)_2_. For anaerobic growth, cultures were grown in the dark without shaking, essentially as described previously (Oren *et al.,* 2014). OD_600_ was recorded with a Spectronic 200 (ThermoFisher Scientific, Waltham, MA) test tube reader. Growth rates were calculated by using the slope of a line of regression calculated from early to mid-log phase.

### 2.2. Enzyme purification

To purify β-galactosidase, cultures of *Halobacterium* sp. NRC-1 (pRK42) (Karan *et al.,* 2013) were grown to late-log phase (OD_600_ ∼ 1.0), harvested, resuspended in binding buffer (20 m*M* phosphate buffer, 2.0 *M* NaCl, 10% (v/v) glycerol, 30 m*M* imidazole, pH 7.4), and lysed by sonication (Model 50 Sonic Dismembrator, ThermoFisher Scientific, Waltham, MA). Cell debris was removed by centrifugation (25,000*g,* 4°C, 10 min) in an Eppendorf 5417C centrifuge, and resulting crude extract was filtered through a 0.2 μm polyethersulfone filter (ThermoFisher Scientific, Waltham, MA). The proteins were purified by using Nickel HisTrap HP Columns (GE Healthcare, Chicago, IL), equilibrated, and washed with binding buffer, and eluted sequentially with 2 *M* NaCl, 100 m*M* phosphate buffer (pH 7.4) supplemented with 40, 80, 100, 200, and 500 m*M* imidazole.

Column fractions were tested for activity with *o*-nitrophenyl-β-D-galactopyranoside (ONPG, ThermoFisher Scientific, Waltham, MA). Active fractions were combined and protein concentration estimated by absorption at 280 nm with a Shimadzu UV-1601 spectrophotometer (Shimadzu Corporation, Kyoto, Japan). To confirm purity, the protein was analyzed by 10% SDS-PAGE followed by staining with Coomassie Blue (Millipore Sigma, Billerica, MA).

### 2.3. Enzyme inhibition

The inhibitory properties of Mg^2+^ and ClO_4_^−^ ions on the β-galactosidase enzyme were tested through steady-state kinetics with a Shimadzu UV-1601 spectrophotometer. The assay solution containing 10 μg/mL enzyme was preincubated at 50°C followed by addition of ONPG at three different concentrations (1, 2.5, and 5 m*M*), and absorption was recorded at 420 nm. *V*_0_, the Michaelis constant (*K*_m_), as well as *V*_max_, were determined with UV Probe ver. 4.23 software (Shimadzu Corporation, Kyoto, Japan). *k*_cat_ was calculated from given values of *K*_m_ and *V*_max_. After the kinetic experiments were recorded, Lineweaver-Burk plots for each concentration of the inhibitory ion were used to determine whether the inhibition was competitive or noncompetitive. *K*_I_ was determined by using Dixon plots, which use the reciprocal velocity of multiple steady-state kinetic experiments at multiple substrate and inhibitor concentrations to extrapolate the concentration of inhibitor that results in half-maximal inhibition (Yoshino and Murakami, 2009). All experiments were run in triplicate and averages used for kinetic analysis.

## 3. Results

### 3.1. Effects of perchlorate salts on haloarchaeal growth

**FIG. 1.**
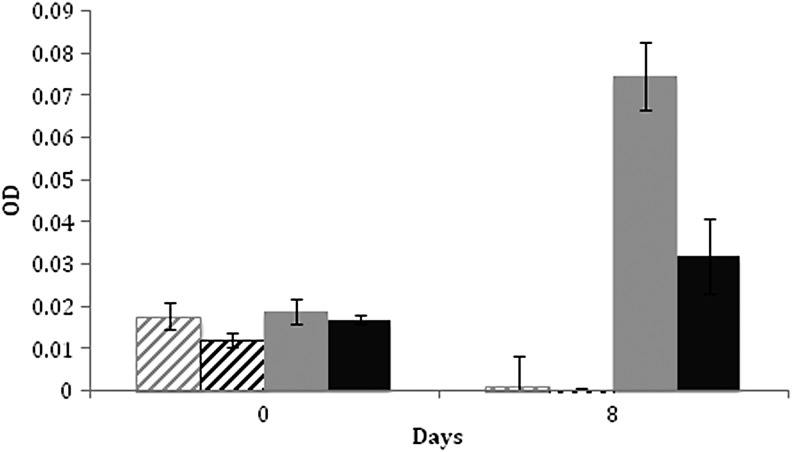
Anaerobic growth assay of *Halorubrum lacusprofundi* and *Halobacterium* sp. NRC-1. Graphs show the anaerobic growth of *H. lacusprofundi* (solid bars) and *Halobacterium* sp. NRC-1 (slashed bars) in the presence (gray) and absence (black) of 40 m*M* NaClO_4_ 0 or 8 days after inoculation. Error bars indicate standard deviation (*n* = 3).

We first sought to determine the effect of the perchlorate anion on anaerobic growth of the Antarctic cold-adapted halophile *H. lacusprofundi,* employing *Halobacterium* sp. NRC-1 as a control. *Halorubrum lacusprofundi* cultures tested in the absence of oxygen in the dark showed that the cold-adapted species is able to grow anaerobically in media supplemented with 40 m*M* perchlorate (Fig. 1). In contrast, the model mesophilic halophile, *Halobacterium* sp. NRC-1, was found to be incapable of anaerobic growth with perchlorate. These findings confirmed that certain haloarchaea are capable of utilizing perchlorate as a terminal electron acceptor, whereas others are not.

**FIG. 2.**
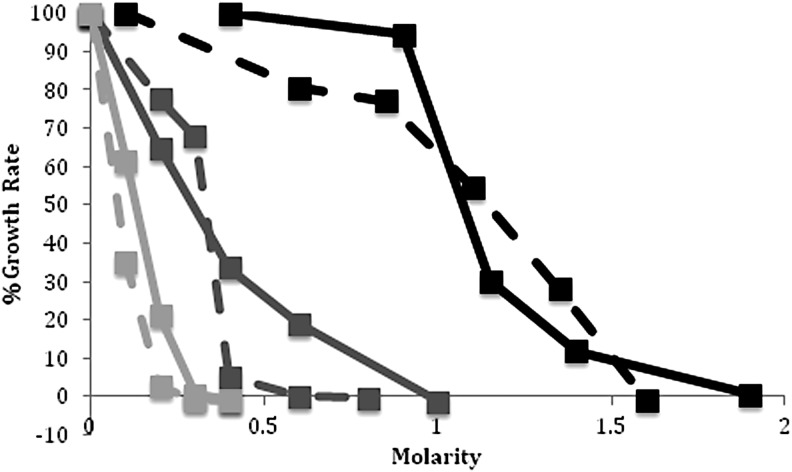
Growth rates of *Halorubrum lacusprofundi* and *Halobacterium* sp. NRC-1 in the presence of added MgCl_2_, NaClO_4_, or Mg(ClO_4_)_2_. Graphs show the percent growth rate for *Halobacterium* sp. NRC-1 (dashed) and *H. lacusprofundi* (solid) when various concentrations of MgCl_2_ (black), NaClO_4_ (dark gray), or Mg(ClO_4_)_2_ (light gray) were added to CM^+^ medium for *Halobacterium* sp. NRC-1 and ATCC 1682 for *H. lacusprofundi*.

To determine the inhibitory effects of higher concentrations of perchlorate, growth was monitored aerobically with up to 1 *M* sodium perchlorate, and an equivalent amount of chloride ions removed to maintain unchanged salinity. *Halorubrum lacusprofundi* growth rate was found to steadily decline with perchlorate concentrations from 0.2 to 1 *M* (Fig. 2). A 50% decrease in growth rate was observed at 0.3 *M* ClO_4_^−^, and complete cessation of growth occurred at 1 *M*. The highest concentration at which a measureable growth rate was recorded was 0.8 *M* ClO_4_^−^. For *Halobacterium* sp. NRC-1, growth rate decreased 30% with 0.3 *M* ClO_4_^−^, with a rather sudden reduction in growth rate from 0.3 to 0.4 *M* (Fig. 2). No measurable growth was observed at perchlorate concentrations of 0.4 *M* or higher.

Next, we determined the effects of magnesium perchlorate on growth rate of the two halophiles, by replacing an equivalent concentration of sodium chloride in their media, with the total anion concentration unchanged. Growth reduction by 50% was observed with 0.12 *M* Mg(ClO_4_)_2_ for *H. lacusprofundi* and 0.07 *M* for *Halobacterium* sp. NRC-1 (Fig. 2). Cessation of growth was observed with 0.3 *M* Mg(ClO_4_)_2_ for *H. lacusprofundi* and 0.2 *M* for *Halobacterium* sp. NRC-1. These results indicate a slightly greater inhibition for the divalent cation compared to the monovalent cation at equivalent perchlorate concentration, for example, 50% inhibition with 0.12 *M* magnesium perchlorate (equivalent to 0.24 *M* perchlorate ions) versus 0.3 *M* sodium perchlorate for *H. lacusprofundi*.

To discriminate the effects of magnesium cations and perchlorate anions on haloarchaeal growth, we also determined the effects of increasing cationic Mg^2+^ concentration on growth by addition of MgCl_2_ to the growth media. *Halorubrum lacusprofundi* retains a substantial growth rate up to 0.9 *M* Mg^2+^ and shows a 40% decrease in the growth rate with 1.2 *M* Mg^2+^ (Fig. 2). The growth rate drops 80% with 1.4 *M* Mg^2+^, and this is the highest concentration at which any measurable growth was observed. *Halobacterium* sp. NRC-1 under similar conditions also showed similar tolerance to increased Mg^2+^, with 50% reduction at 1.1 *M* MgCl_2_ and complete inhibition of growth at 1.6 *M* MgCl_2_ (Fig. 2). These findings show that the inhibitory effect of perchlorate anions is dominant over magnesium cations for haloarchaea.

### 3.2. Effects of ions on enzyme activity

**FIG. 3.**
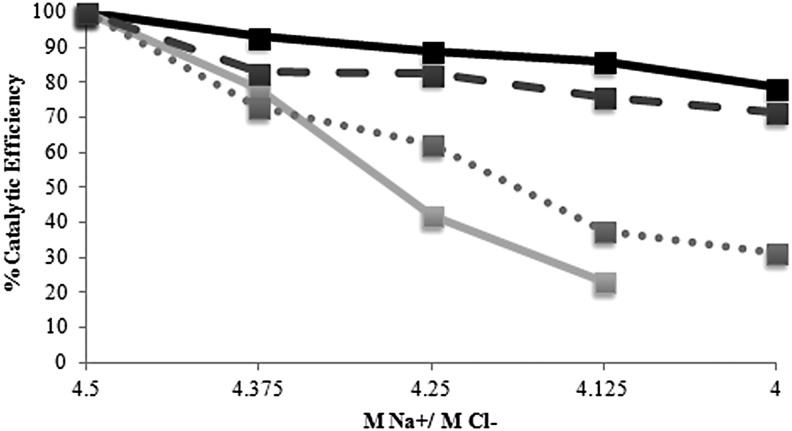
Percent catalytic efficiency of β-galactosidase (pRK42) under the influence of different ions. Kinetic experiments were performed in various solutions using 10 μg/mL of enzyme per reaction at 50°C with ONPG concentrations of 1, 2.5, and 5 m*M*. Various molarities of NaCl were tested with no added magnesium or perchlorate (black). Ratios of sodium and magnesium chloride were tested (dotted gray). Ratios of sodium chloride and perchlorate were tested (dashed gray). Ratios of sodium chloride to magnesium perchlorate were tested (light gray). Concentrations higher than 0.18 *M* Mg(ClO_4_)_2_ showed no activity.

To determine the effects of magnesium perchlorate on a halophilic enzyme, we performed inhibition assays on the *H. lacusprofundi* β-galactosidase enzyme by replacing NaCl with either sodium or magnesium perchlorate in the reaction buffer (Fig. 3). Replacing 0.5 *M* Cl^−^ with ClO_4_^−^ using sodium perchlorate resulted in 30% decrease, while replacing 0.5 *M* Na^+^ with 0.25 *M* Mg^2+^ using magnesium chloride resulted in 70% decrease in catalytic efficiency. With Mg(ClO_4_)_2_, catalytic efficiency was decreased 70% at 0.18 *M*, and no measurable activity was observed at 0.23 *M* and higher (Fig. 3). These findings showed inhibitory effects of both ions Mg^2+^ and ClO_4_^-^ together or separately, which were significantly greater than that of reducing sodium chloride concentration alone.

**FIG. 4.**
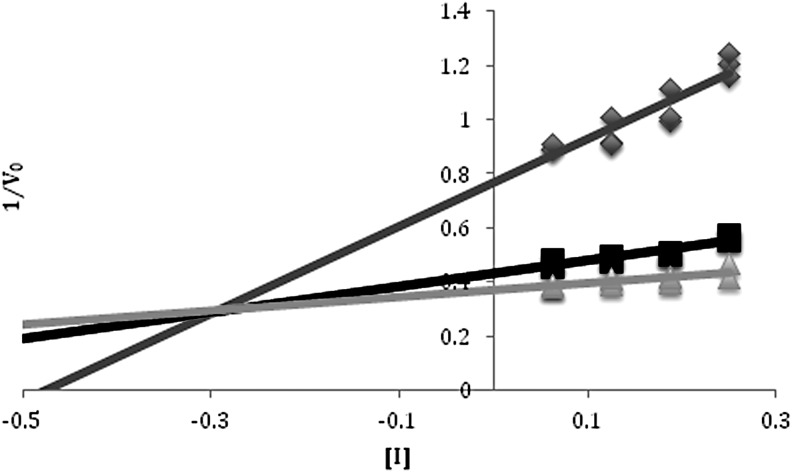
Dixon plot of MgCl_2_ inhibition of *Halorubrum lacusprofundi* β-galactosidase. The inverse of *V*_0_ for each ONPG concentration was plotted versus the concentrations of MgCl_2_. Where the lines intersect is the –*K*_I_. Kinetics were performed at 5 m*M* (dark gray), 2.5 m*M* (black), and 1 m*M* (light gray) ONPG in 0.0625–0.25 *M* MgCl_2_ and 4.375–4 *M* NaCl at 50°C. Each reaction had a total volume of 500 μL with 10 μg/mL of enzyme. The value determined for *K*_I_ was 0.3 *M*.

**FIG. 5.**
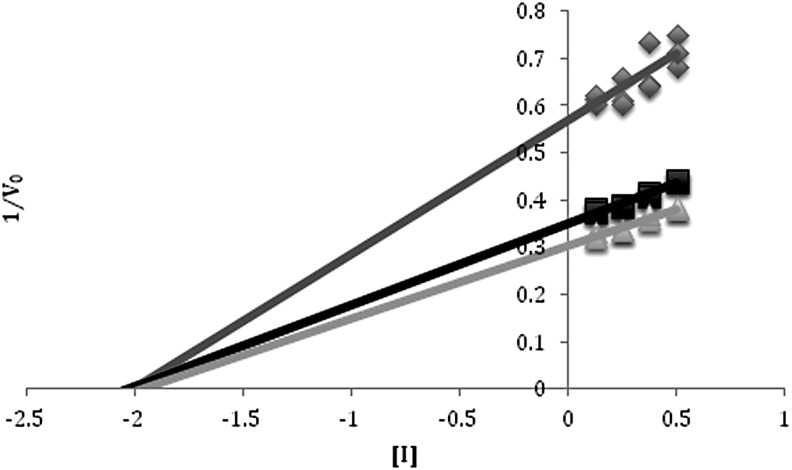
Dixon plot of NaClO_4_ inhibition of *Halorubrum lacusprofundi* β-galactosidase. The inverse of *V*_0_ for each ONPG concentration was plotted versus the concentrations of NaClO_4_. Where the lines intersect with each other and the *x* axis is the –*K*_I_. Kinetics were performed at 5 m*M* (dark gray), 2.5 m*M* (black), and 1 m*M* (light gray) ONPG in 0.125–0.5 *M* NaClO_4_ and 4.375–4 *M* NaCl at 50°C. Each reaction had a total volume of 500 μL with 10 μg/mL of enzyme. The value determined for *K*_I_ was 2 *M*.

**FIG. 6.**
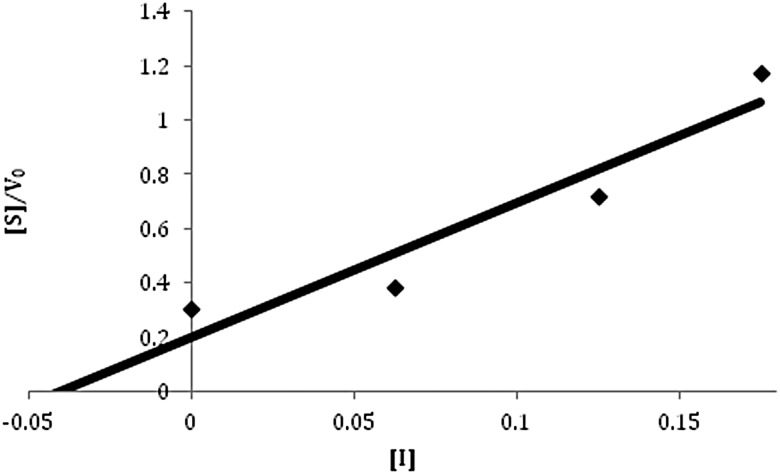
Plot of Mg(ClO_4_)_2_ kinetics. The Lineweaver-Burk slope for each concentration of Mg(ClO_4_)_2_ was plotted against the concentration of the inhibitor using the alternative Dixon plot equation: $\frac { 1 }  { V } = { \frac { { K_m } }  { { V_ { max } } } } \left( { \frac { 1 }  { { \left[ { \rm { S } } \right] } } } \right) \left( { 1 + { \frac { \left[ { \rm { I } } \right] }  { { K_I } } } } \right) + \frac { 1 }  { { { V_ { max } } } } \left( { 1 + { \frac { \left[ \rm I \right] }  { { K_I } } } } \right)$. A linear regression was performed, and the *x* intercept represents the –*K*_I_. Kinetics were performed at 5, 2.5, and 1 m*M* ONPG in 0.0625–0.225 *M* Mg(ClO_4_)_2_ and 4.375–4.05 *M* NaCl at 50°C. Each reaction had a total volume of 500 μL with 10 μg/mL of enzyme. The value determined for *K*_I_ was 0.04 *M*.

To characterize the nature of inhibition, kinetic analysis was carried out for β-galactosidase reactions with added MgCl_2_, NaClO_4_, and Mg(ClO_4_)_2_. Dixon plots for magnesium chloride showed behavior as a competitive inhibitor with a *K*_I_ of 0.3 *M* for Mg^2+^ (Fig 4). The Dixon plot for sodium perchlorate showed noncompetitive inhibition with a considerably higher *K*_I_ of 2 *M* NaClO_4_ (Fig 5). When the two inhibitors were combined through the addition of Mg(ClO_4_)_2_ to the reaction buffer, mixed inhibition characteristics were observed from the Dixon plot (Fig. 6), with a *K*_I_ of 0.04 *M* for Mg(ClO_4_)_2_, a considerably more potent effect than when added separately.

## 4. Discussion

We investigated the effects of sodium and magnesium perchlorate salts prevalent on Mars on growth and enzyme function of a cold-adapted extreme halophile from Antarctica. *Halorubrum lacusprofundi* was found to grow anaerobically in low concentrations of perchlorate and was inhibited at considerably higher concentrations, with more sensitivity to magnesium perchlorate compared to sodium perchlorate (50% inhibition at 0.3 *M* sodium perchlorate versus 0.1 *M* for magnesium perchlorate). Similar results were obtained for inhibition of the purified *H. lacusprofundi* β-galactosidase enzyme, with greater enzyme activity reduction for magnesium perchlorate compared to sodium perchlorate (50% inhibition with 0.88 *M* sodium perchlorate and 0.13 *M* magnesium perchlorate). Interestingly, steady-state kinetic analysis showed that magnesium ions act as a competitive inhibitor for the enzyme, while perchlorate ions act as a noncompetitive inhibitor, with magnesium perchlorate acting as a mixed inhibitor.

According to spectroscopic analysis, martian soil contains toxic sodium and magnesium perchlorates, leading to the suggestion that Mars may not be hospitable to any form of life as we know it (Mattie *et al.,* 2006; Wadsworth and Cockell, 2017). While there is a considerable range of concentrations of perchlorate ions on the surface of Mars, the high concentration has been estimated to be 0.1225 *M* for the sodium salt and 0.0532 *M* for the magnesium salt, based on weight/volume (Ojha *et al.,* 2015). When dissolved into soil, millimolar concentrations are expected (*e.g.,* 1.4 m*M* Na^+^, 3.3 m*M* Mg^2+^, and 2.4 m*M* ClO_4_^-^) (Hecht *et al.,* 2009). These concentrations are well below the 50% inhibition rates measured for growth of both halophiles. The degree of inhibition of both growth and enzyme activity indicates that the expected concentrations of perchlorate salts on Mars would by themselves be unlikely to preclude success of extreme halophiles such as *H. lacusprofundi* on the Red Planet.

Compared to non-halophilic archaea, extreme halophiles may be able to tolerate higher concentrations of the perchlorate ions. For example, *Methanobacterium articum* M2 VKM B-2372, *Methanobacterium veterum* MK4 VKM B-2440, and a *Methanosarcina* sp. exhibited 80% reduction in methanogenesis with addition of just 9.8 m*M* NaClO_4_ (Shcherbakova *et al.,* 2015), 30 times lower concentration than tolerated by *H. lacusprofundi* and *Halobacterium* sp. NRC-1. However, some methanogens metabolize up to 0.06 *M* Mg(ClO_4_)_2_ when grown on Mars soil simulant (Kral *et al.,* 2004), with successful selection of *Methanothermobacter wolfeii* and *Methanosarcina barkeri* strains able to tolerate up to 0.3 *M* perchlorate (Kral *et al.,* 2016). These findings support the notion that halophilic and methanogenic archaea may indeed potentially survive on Mars, with growth possibly stimulated by perchlorate reduction.

Although its mechanism of action has not been extensively studied, perchlorate toxicity in microorganisms likely results from oxidative stress. Halophiles are known to be highly resistant to some of the damaging effects of oxidative stress, resulting from novel repair genes in their genomes. For example, double-stranded DNA damage repair genes that permit survival from desiccation and ionizing radiation damage have been extensively tested in the model *Halobacterium* sp. NRC-1 (DeVeaux *et al.,* 2007; Karan *et al.,* 2014). In addition, a large number of other repair systems, including novel superoxide dismutases, have been reported in the genomes of extreme halophiles (May and Dennis 1990; Joshi and Dennis 1993; Capes *et al.,* 2011). Additional studies are required to fully understand the genetic basis of the high perchlorate tolerance in halophiles (Oosterkamp *et al.,* 2011).

A recent report indicated that UV radiation enhances the toxicity of perchlorates in certain bacteria. When *Bacillus subtilis* was grown with levels of perchlorate expected on Mars, it was able to grow without any inhibition, but when those concentrations of perchlorate were combined with UV, the cells lost viability (Wadsworth and Cockell, 2017). Since halophiles display considerably more UV and perchlorate tolerance than most bacteria such as *B. subtilis,* the combinatorial effect may not be as debilitating (DasSarma *et al.,* 2001; McCready *et al.,* 2005). *Halobacterium* sp. NRC-1 can tolerate upward of 100 J/m^2^ of UVC radiation as a result of efficient light and dark repair systems, and may have better capability to tolerate a combination of damaging effects with perchlorate. Preliminary results indicate smaller or negligible effects with a combination of UV and perchlorate (our unpublished results).

Mg^2+^ ions were also inhibitory for growth of both *H. lacusprofundi* and *Halobacterium* sp. NRC-1, albeit less potent than perchlorate. Growth media for *H. lacusprofundi* contains 0.4 *M* magnesium compared to 0.08 *M* for *Halobacterium* sp. NRC-1, reflecting the relatively higher magnesium concentration in Deep Lake where the cold-adapted species was isolated, compared to Great Salt Lake and similar thalassic environments where the mesophile has been found. Addition of Mg^2+^ up to nearly 1 *M* concentration did not have any significantly deleterious effect. However, at even higher concentrations, above 1 *M* Mg^2+^, growth of both halophiles could be completely inhibited, with the growth rate for the cold-adapted species declining more steeply. Additional studies are needed to understand high magnesium ion tolerance and potential involvement of heavy metal efflux systems (Nies, 2003; Srivastava and Kowshik, 2013).

When looking at the effect on purified *H. lacusprofundi* β-galactosidase enzyme, Mg^2+^ acted as a competitive inhibitor with a *K*_I_ of 0.3 *M*. This *K*_I_ is lower than the concentration of Mg^2+^ in the medium, suggesting that, when the cation is added, it does not increase the concentration within the cell and has little effect on the enzyme function. Divalent ions have been shown to positively and negatively affect enzymatic reactions (Mordasini *et al.,* 2003). The higher charge and smaller size of Mg^2+^ ions compared to sodium ions may contribute to the inhibitory effect on the enzyme through potential competition with other cation binding sites and disruption of the hydration shell. Not surprisingly, β-galactosidase is typical of halophilic proteins in having a large net surface charge and the potential of binding many positively charged Mg^2+^ ions (Kennedy *et al.,* 2001). Mg^2+^ may also interfere with binding of zinc in β-galactosidase, although there is no known function for zinc ions in the *T. thermophilus* enzyme (Hidaka *et al.,* 2002).

Perchlorate is known to be a chaotropic anion and has the potential to act as a strong denaturing agent (Zhang and Cremer, 2010). However, the anion is a noncompetitive inhibitor with a large *K*_I_ of 2 *M*, indicating that it is a weak inhibitor of β-galactosidase. The denaturing and/or oxidative properties of perchlorate could explain the noncompetitive inhibitory effects on the enzyme. Perchlorate is also an effective salting-out anion, but at the concentrations tested, no protein precipitation could be observed. When compared to the weak enzyme inhibitory effect, growth of both *Halobacterium* sp. NRC-1 and *H. lacusprofundi* is strongly inhibited by perchlorate. This may result from facile uptake of ClO_4_^−^ ions and other effects, for example, oxidative damage. Protein negative charges may repel the negatively charged perchlorate ions such that they may not interfere with enzyme function except at very high concentrations.

The observed effects of Mg(ClO_4_)_2_ suggest synergistic action of magnesium and perchlorate ions to inhibit the growth of both *H. lacusprofundi* and *Halobacterium* sp. NRC-1, as well as β-galactosidase enzyme activity. Both *H. lacusprofundi* and *Halobacterium* sp. NRC-1 exhibited 50% growth inhibition at 0.1 *M* Mg(ClO_4_)_2_, which is a lower concentration than either magnesium or perchlorate ions alone. However, perchlorate has more detrimental effects on growth than magnesium ions, suggesting that halophilic cells are able to keep excess Mg^2+^ outside of cells, but the ClO_4_^−^ ions are able to enter and potentially cause damage to DNA (Bauer, 2002). When looking at the purified enzyme, the main effect is competitive inhibition from the Mg^2+^ ions as well as the lesser effects from noncompetitive inhibition from ClO_4_^−^, resulting in mixed inhibition from Mg(ClO_4_)_2_ with a *K*_I_ of 0.04 *M*.

In summary, the presence of Mg(ClO_4_)_2_ and NaClO_4_ on Mars, while inhibitory for most microorganisms, likely would not preclude the growth and function of haloarchaea, such as *H. lacusprofundi,* tested in this study. Perchlorate ions do inhibit the growth of haloarchaeal cells, and magnesium ions can cause inhibition of a halophilic enzyme, but with complete inhibition occurring at concentrations much higher than those reported on Mars.

## Abbreviation Used

ONPG: *o*-nitrophenyl-β-D-galactopyranoside
